# Transcriptomic Profiling in Aged Mice Reveals an Association Between Sevoflurane Anesthesia and Neurocognitive Dysfunction

**DOI:** 10.1007/s10571-026-01677-y

**Published:** 2026-01-30

**Authors:** Naiqi Jiang, Junjie Zou, Meiling Tian, Zaibin Jing, Wanting Ding, Lei Wang, Hongzhe Bei, Cuicui Yu

**Affiliations:** 1 School of Anesthesiology, Shandong Second Medical University, Weifang, 261053 China; 2 Department of Neurology, Yantai Penglai People’s Hospital, Yantai, 264117 China; 3 Department of Neurology, The Affiliated Yantai Yuhuangding Hospital of Qingdao University, No.20 Yuhuangding East Road, Yantai, 264000 China; 4 The Second School of Clinical Medicine of Binzhou Medical University, Binzhou Medical University, Yantai, 264003 China; 5 Department of Anesthesiology, The Affiliated Yantai Yuhuangding Hospital of Qingdao University, No.20 Yuhuangding East Road, Yantai, 264000 China; 6 Department of Neurology, Baogang Hospital of Inner Mongolia, Baotou, 014010 China

**Keywords:** Sevoflurane, Neurocognitive disorders, Transcriptomics, Aged mice

## Abstract

Sevoflurane is the most commonly used inhalational anesthetic in clinical work. Exposure to sevoflurane can induce cognitive dysfunction, particularly in the elderly. Transcriptomics aims to investigate diseases at the genetic level, identify enriched pathways and confirm action targets. This study investigates the mechanisms of sevoflurane-induced neurocognitive dysfunction in aged mice through transcriptomic analysis, aiming to establish a theoretical basis for the prevention and treatment of cognitive impairment. Eighteen-month-old aged mice were anesthetized with sevoflurane for 4 h. Hippocampal tissues were collected for transcriptomic analysis following the water maze test. Morris water maze (MWM) testing revealed that sevoflurane-exposed mice exhibited significantly longer escape latencies (*b* = 8.02, 95% CI [2.89, 13.16]) and fewer platform crossings (*U* = 65, *Z* = -2.018, *P* = 0.044) compared to controls. Transcriptomic profiling identified 148 differentially expressed genes (DEGs) associated with cysteine and methionine metabolism. KEGG enrichment analysis confirmed that the Cysteine and methionine metabolism pathway was the most significantly altered (FDR = 0.006). Sevoflurane may impair hippocampal function and our transcriptomic data suggest a potential association with dysregulation of metabolic pathways, particularly cysteine and methionine metabolism, which may contribute to cognitive impairment. Sevoflurane anesthesia is linked to transcriptomic alterations in the brains of aged mice, primarily affecting cysteine and methionine metabolism. Sevoflurane anesthesia may suppress widespread gene expression and could lead to the deactivation of functional networks in the brains of aged mice. The raw RNA-seq data have been deposited in the China National GeneBank Database (CNGBdb) under accession number CNP0007894.

**Supplementary Information:**

The online version contains supplementary material available at 10.1007/s10571-026-01677-y.

## Introduction

According to recent studies, the goal of LCoGS 2030 is to perform 5,000 surgeries per 100,000 people each year (Meara et al. 2015). The incidence of neurocognitive disorders is approximately 15%-25%, with the majority being elderly individuals (Geng et al. 2017). Compared to young patients, patients over 65 years old are more prone to cognitive dysfunction after anesthesia (Berian et al. 2018). Neurocognitive dysfunction, a central nervous system complication associated with anesthesia, manifests as memory impairment, reduced learning capacity, altered cognition, diminished quality of life, increased complications, and higher morbidity and mortality rates (Sun et al. 2024; Wang et al. 2024b). In recent years, studies have found that sevoflurane can cause neurotoxicity, especially neurocognitive dysfunction (Yang et al. 2024b). Studying neurocognitive dysfunction caused by anesthesia is of great significance for improving the quality of life of patients.

Transcriptomics, a key discipline in mechanistic research, enables comprehensive analysis of disease-related gene expression profiles, identification of critical pathways, and development of novel therapeutic strategies (Roychowdhury and Chinnaiyan 2016). Transcriptomic sequencing has greatly enhanced our understanding of the mechanism of anesthesia-induced neurocognitive dysfunction and the role of anesthetic drugs in promoting neurodegenerative diseases (Yang et al. 2024a; Xie et al. 2024). By analyzing key genes and signaling pathways involved in complex neurocognitive disorders, transcriptomics offers insights for preventing and treating cognitive impairment in patients.

In recent years, numerous researchers have validated the association between sevoflurane and neurocognitive impairment through animal experiments, primarily focusing on four key pathways: synaptic dysfunction, neuroinflammation, oxidative stress with ferroptosis, and neuronal apoptosis (Tao et al. 2014; Liu et al. 2021; Zhou et al. 2023b; Xu et al. 2023). Beyond transcriptomics, researchers employed chemogenetics and photogenetics techniques, combined with advanced methods such as multi-channel recording in vivo and electroencephalogram, to confirm the crucial role of sodium leakage channels in maintaining the activity of paraventinal neurons in the thalamus and promoting the recovery process after sevoflurane anesthesia, providing a new perspective (Xia et al. 2024; Wu et al. 2024). For specific intervention measures, such as inhibiting neuroinflammation caused by phosphorylated expression of Tau protein or inhibiting mitochondrial-mediated hippocampal neuronal apoptosis through mitochondrial autophagy and other mechanisms (Zhu et al. 2022; Zhou et al. 2023a).

Sevoflurane-induced neurocognitive dysfunction in aged mice is a multifaceted process involving complex interlinked pathways. Current research predominantly explores isolated molecular targets, signaling cascades or single therapeutic agents, failing to provide a holistic transcriptomic explanation of these pathways. Moreover, the mechanisms of action of key molecules remain unclear. Building on existing studies, this research aims to systematically investigate the transcriptomic mechanisms of sevoflurane-induced neurocognitive dysfunction in aged mice, with the goal of establishing a theoretical foundation for preventing and treating such cognitive deficits.

**Fig. 1:**
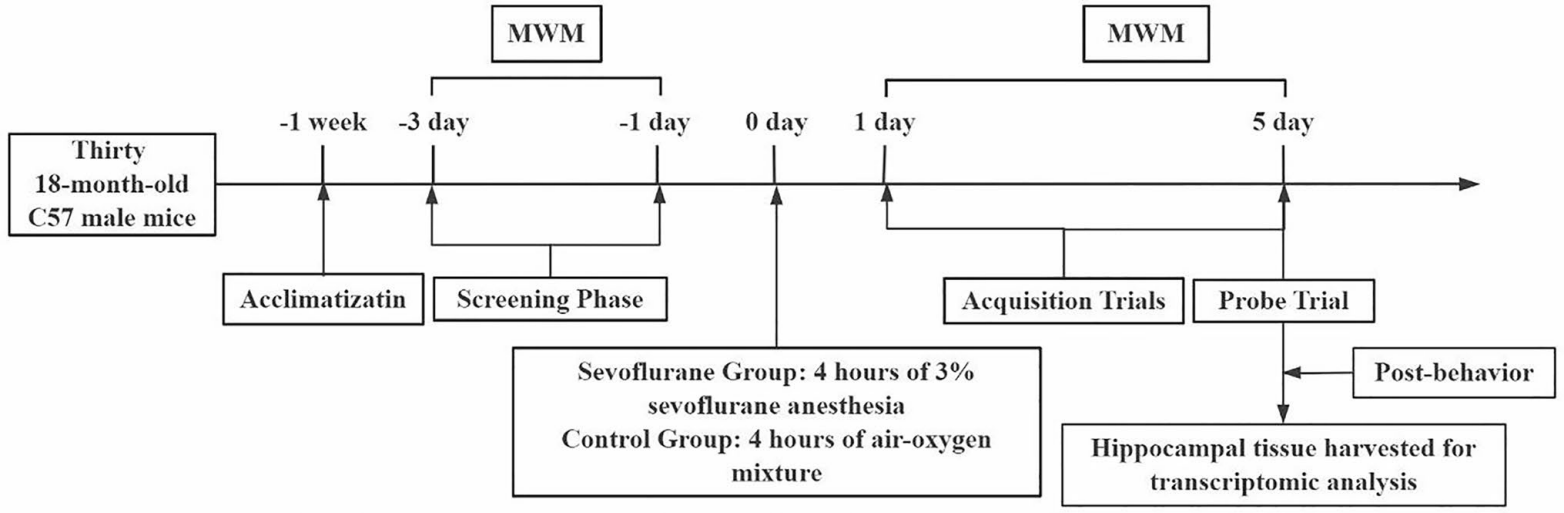
Schematic timeline of the experimental procedure

18-month-old C57 male mice were acclimatized for one week. Following acclimatization, mice underwent a three-day Morris water maze (MWM) screening phase to select eligible subjects for the study. The enrolled mice were then randomly assigned to receive either 4 h of sevoflurane anesthesia or a control air-oxygen mixture. Twenty-four hours post-anesthesia, the MWM test was conducted, consisting of a 5-day acquisition training phase followed by a probe trial on day 6. Immediately after the behavioral tests, six mice were randomly selected from each group for hippocampal tissue collection and subsequent transcriptomic sequencing and analysis (See Fig. 1).

## Materials and Methods

Sample sizes were determined based on effect sizes observed in prior similar studies (Dai et al. 2024; Wang et al. 2024b) and met or exceeded common standards in transcriptomic Mechanisms of sevoflurane-induced neurocognitive dysfunction in Aged Mice for detecting significant differences.

All experiments were conducted in accordance with the ARRIVE Essential 10 guidelines to ensure rigorous and transparent reporting. Detailed information on study design, sample size justification, randomization, blinding, statistical methods and experimental procedures can be found in the respective sections of the manuscript. The completed ARRIVE 2.0 checklist is provided as part of the submission. The completed ARRIVE guidelines checklist is available in the Supplementary Materials.

### Experimental Animals

Thirty 18-month-old C57 male mice (Shye et al. 1994) were purchased from Changzhou Cavens Laboratory Animal Co.,Ltd. The mice were specific pathogen-free (SPF) and housed under SPF conditions throughout the study. All experimental procedures were approved by the Ethics Committee of Yantai Yuhuangding Hospital (Approval No: 2025 − 121) and complied with national guidelines for the management and use of experimental animals. These mice were experimentally naïve and had not undergone any procedures prior to the start of this study. Measures to ensure welfare included monitored anesthesia, controlled housing conditions and postoperative care. The mice were housed in an environment maintained at (24 ± 2)℃ and 50% humidity, with five mice per cage. The core body temperature of the mice was continuously monitored and maintained at (37 ± 0.5)°C with a feedback-controlled heating system. All cages were identical and a normal light-dark cycle was maintained. Adequate food and water were provided and the mice were acclimatized for one week prior to experiments.

### Grouping and Sevoflurane Anesthesia

The 30 aged mice were randomly assigned to either the sevoflurane anesthesia group or the control group (*n* = 15 per group). The randomization sequence was generated using a computer-based random number table by an independent researcher (J.Z.) who was not involved in the subsequent animal handling or behavioral testing. The group allocation was concealed in sealed envelopes until the commencement of the anesthesia intervention.

Due to the nature of the intervention, the researcher preparing the anesthesia (M.T.) was aware of the group assignments but was not involved in any subsequent experimental procedures or outcome assessments.

The aged mice were placed in a sealed transparent plastic chamber lined with dry bedding material. The temperature was maintained at (24 ± 2)℃ throughout the anesthesia procedure. The plastic chamber had two ports: one connected to oxygen and the other to sevoflurane. For the sevoflurane group, anesthesia was induced with 5% sevoflurane combined with an air-oxygen mixture (gas flow rate: 2 L/min) for 3–5 min until the righting reflex and tail pinch reflex disappeared.The sevoflurane concentration was then adjusted to 3% using a vaporizer and 3% sevoflurane with the air-oxygen mixture (gas flow rate: 2 L/min) was continuously delivered for 4 h.The control group received only the air-oxygen mixture (gas flow rate:2 L/min) under the same conditions and duration.

After anesthesia, both groups were continuously supplied with the air-oxygen mixture (gas flow rate: 2 L/min) until the righting reflex resumed and the mice regained free movement. The mice were then returned to separate cages according to their groups and continued to be housed under standard conditions.

### Animal Inclusion and Exclusion Criteria

Inclusion Criteria: (1) Mice that successfully completed the 3-day acquisition trial during the screening phase; (2) Mice exhibiting normal health status, without signs of systemic illness or severe sensorimotor deficits prior to anesthesia.

Exclusion Criteria: (1) Remaining motionless (floating) for more than 20s in any trial; (2) Failing to find the hidden platform within 60s in all four trials on any given day, indicating a possible lack of motivation or severe sensorimotor deficits; (3) Displaying obvious signs of illness or injury unrelated to the experimental intervention. All 30 initially enrolled mice successfully completed the study protocol without meeting any exclusion criteria, and thus all were included in the final analysis.

### Morris Water Maze Test

The Morris water maze (MWM) test was used to assess cognitive function. The circular pool (diameter: 120 cm, height: 50 cm) was divided into four equal quadrants (I, II, III, IV) by four equidistant markings on the pool wall. A transparent platform (diameter: 6 cm) was placed in the center of quadrant III, submerged 2 cm below the water surface. The pool was filled with water maintained at 24℃ and dyed opaque white using edible titanium dioxide. An automated video tracking system above the pool recorded movement trajectories, total distance traveled, escape latency, time spent in each quadrant, and the number of platform crossings.

The researchers conducting the behavioral tests and scoring (N.J., Z.J. and W.D.) were blinded to the group allocation of the mice.

Screening phase: A 3-day acquisition trial was conducted for all 30 mice to confirm baseline consistency. Mice showing abnormal performance (immobility or extreme latency) were excluded and the remaining mice proceeded to formal testing.

Acquisition trials: At a fixed time daily, mice were placed into the water facing the pool wall at one of four randomized entry points and allowed to swim freely until locating the hidden platform. The automated system tracked escape latency (time to reach the platform) and swimming paths. If the mouse failed to locate the platform within 60s, the operator guided it to the platform and ensured a 15-second stay, with the escape latency recorded as 60 s. Each mouse underwent four trials per day and the average of four trials was calculated as the daily result.

Probe trial: After 5 days of acquisition trials, the hidden platform was removed. Mice were placed into quadrant I and their swimming trajectories and number of crossings over the former platform location were recorded for 60s.

### Transcriptomic Analysis

After behavioral tests, six mice were randomly selected from each group and decapitated. Hippocampal tissues were rapidly dissected on dry ice, rinsed with saline, and stored in liquid nitrogen at -80℃. Hippocampal tissues were collected immediately after behavioral tests. Mice were euthanized by rapid decapitation under deep anesthesia to minimize suffering and ensure tissue quality for RNA sequencing.

Following tissue collection, all hippocampal samples were coded. Personnel responsible for RNA extraction, library preparation, sequencing and the subsequent bioinformatic analysis were blinded to the sample codes corresponding to the experimental or control groups until the final statistical comparisons were completed.

Raw sequencing data were quality-controlled and filtered using fastp (Chen et al. 2018) to obtain clean reads. The filtering criteria were: removal reads containing adapters; removing reads containing more than 10% of unknown nucleotides(N); remove reads consisting only of a single base (e.g., all A bases); removing low quality reads containing more than 50% of low quality (Q-value ≤ 20) bases. Post-filtering, base composition and quality distribution were visualized to confirm high data quality. All 12 samples (*n* = 6 per group) passed these criteria and were retained for analysis.

Total RNA was extracted from samples, followed by rRNA depletion and enrichment of mRNA using mRNA Capture Beads. Purified mRNA was fragmented at high temperature. First-strand cDNA was synthesized from fragmented mRNA using a reverse transcriptase mixture, and second-strand cDNA was synthesized with simultaneous end repair and A-tailing. Adapters were ligated to the cDNA and target fragments were selected. The library was amplified by PCR and subjected to quality control (QC) before sequencing.

Raw sequencing reads were quality-controlled and adapter-trimmed using fastp to generate high-quality clean reads. Ribosomal RNA was removed by alignment to an rRNA database using Bowtie2 (version 2.2.8). Clean reads were then aligned to the mouse reference genome (Ensembl release 113) using HISAT2 2.1.0. Transcript assembly and gene abundance quantification were performed using StringTie v1.3.1 and RSEM software, with expression levels reported in TPM (Transcripts Per Kilobase of exon model per Million mapped reads). Differential expression analysis was conducted using DESeq2 software, with genes meeting the threshold of FDR < 0.05 and |log2FC| > log2(1.5) considered statistically significant. Functional enrichment analysis of GO terms and KEGG pathways was performed using hypergeometric testing with FDR correction.

### Power Calculation

The sample size for this study (*N* = 15 per group for behavior, *N* = 6 per group for transcriptomics) was determined based on two primary considerations. First, we referenced effect sizes and sample sizes commonly reported in prior similar studies investigating sevoflurane-induced cognitive dysfunction in aged rodents using the MWM test (Tao et al. 2014; Zhou et al. 2023b), where groups of *N* = 15 are typically sufficient to detect significant differences in escape latency and platform crossings. Second, for transcriptomic sequencing, a sample size of *N* = 6 per group is widely established (Zheng et al. 2024) and considered robust in the field for achieving reliable detection of differentially expressed genes (DEGs), as it provides sufficient biological replicates to account for inter-individual variability while maintaining statistical power for RNA-seq analysis.

### Statistical Analysis

Statistical analyses were performed using SPSS Statistics version 25. When performing multiple comparisons across groups, data were analyzed using a linear mixed model that included group, time and their interaction as fixed effects, incorporating movement velocity and the percentage of time spent in the center zone as covariates. A random intercept for each mouse was included to account for within-subject correlations across repeated measurements. Due to sample size constraints and to ensure model convergence and statistical power, random slopes were not included in the final model; this limitation is acknowledged in the Discussion section. Normality was assessed using the Shapiro-Wilk test and homogeneity of variance was assessed using Levene’s test. Normality and homogeneity of variance were assumed if *P* > 0.05. Non-normally distributed measurement data were expressed as Median and Interquartile Range [M(P_25_, P_75_)]; group comparisons were performed using the Mann-Whitney *U* test. *P* < 0.05 was considered statistically significant. “N”: biological replicates and “n”: technical replicates.

## Results

### Morris Water Maze Test

#### Spatial Navigation Test (N = 15, n = 4)

**Fig. 2:**
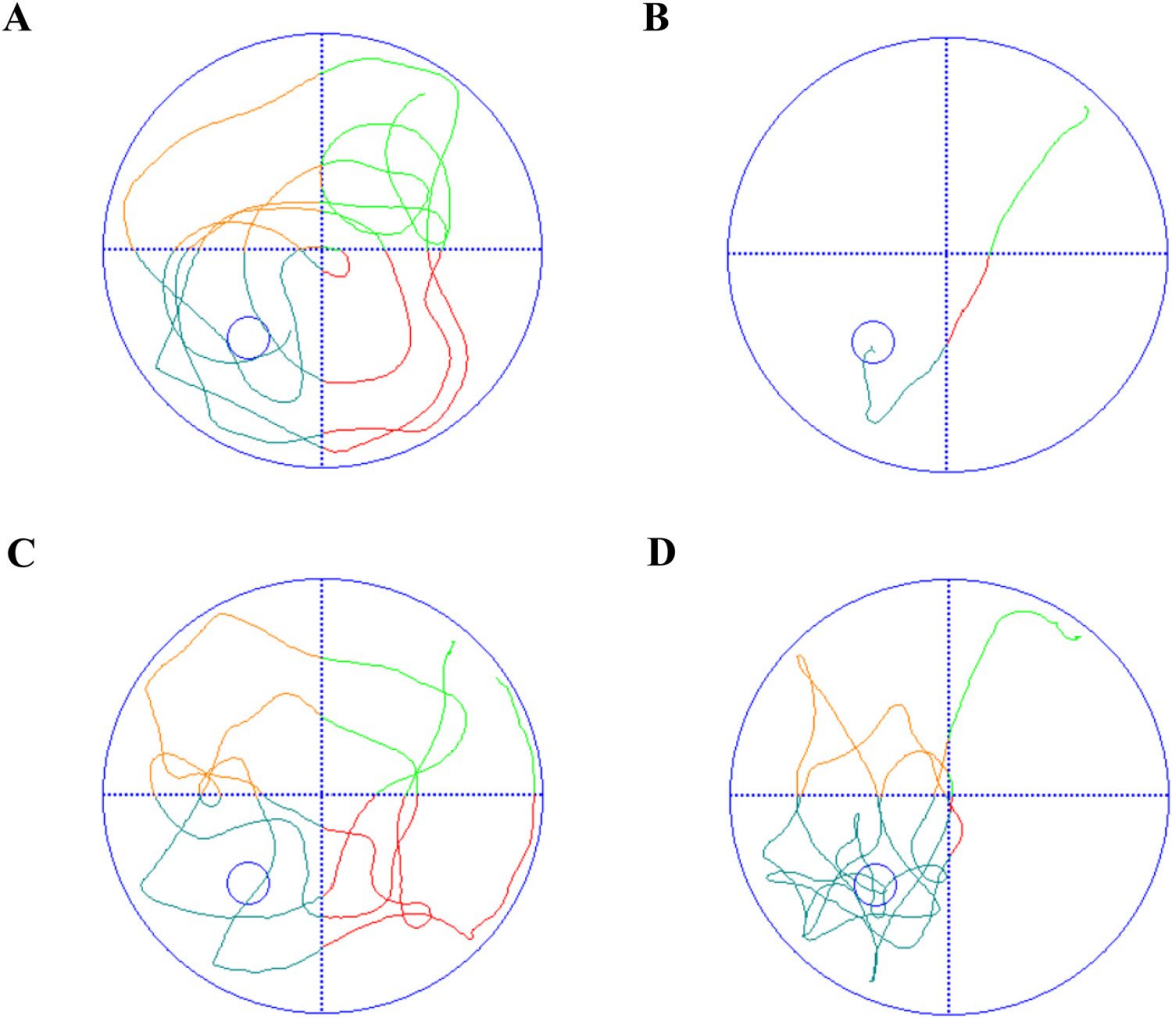
Representative swimming paths from the place navigation task and spatial probe test (*N* = 15). **A**: Swimming path of the experimental group in the place navigation task; **B**: Swimming path of the control group in the place navigation task; **C**: Swimming path of the experimental group in the spatial probe test; **D**: Swimming path of the control group in the spatial probe test

The mixed-model analysis revealed a critical influence of behavioral strategy on spatial learning (Table 1). Specifically, there was a significant main effect of group, with the sevoflurane group showing longer escape latencies than the control group (*b* = 8.02, 95% CI [2.89, 13.16]). A significant negative association was observed between the percentage of time spent in the center zone and escape latency (*b* = -73.93, 95% CI [-97.22, -50.63]). This indicates that for every 1% increase in center time, escape latency decreased by approximately 0.74 s, suggesting that animals with a stronger tendency to explore the center exhibited better cognitive performance. Conversely, movement velocity showed a significant positive association with escape latency (*b* = 0.18, 95% CI [0.11, 0.24]), meaning that for every unit increase in velocity, escape latency increased by an average of 0.18s. This supports the hypothesis that higher movement speed may reflect an inefficient exploration strategy (See Fig. 2A and B).

**Table 1:** Bhavioral strategy on spatial learning (N = 15, n = 4)

Predictor	Estimate (b)	df	t-value	*P*-value	95% CI
Group	8.02	37.05	3.17	0.003*	[2.89, 13.16]
Center Time Percentage	-73.93	59.64	-6.35	< 0.001*	[-97.22, -50.63]
Velocity	0.18	72.96	5.34	< 0.001*	[0.11, 0.24]

Group: The escape latency was longer in the experimental group than in the control group; Center Time Percentage: A negative correlation was observed between the percentage of time spent in the center zone and escape latency in mice; Velocity: movement velocity during the task showed a positive correlation with escape latency. Estimate (*b*): unstandardized regression coefficient (effect size); CI: confidence interval

*Significant, bold text indicates statistical significance (*P* < 0.05); “N”: Biological replicates; “n”: Technical replicates

#### Platform Crossings (N = 15, n = 1)

The MWM tests were performed on 15 mice per group after anesthesia. On Day 5, Shapiro-Wilk normality test results for the experimental group were non-significant (*W* = 0.902, *P* = 0.104), indicating normally distributed data. Shapiro-Wilk test results for the control group were significant (*W* = 0.867, *P* = 0.031), indicating that the data were not normally distributed. Levene’s test for homogeneity of variances on Day 5 scores was non-significant (*F*(1, 28) = 0.972, *P* = 0.333), indicating equal variances. However, since the control group data violated the normality assumption (Shapiro-Wilk *W* = 0.902, *P* = 0.104) while the experimental group met it (Shapiro-Wilk *W* = 0.867, *P* = 0.031), the primary analysis employed the Mann-Whitney *U* test. Results showed the experimental group scored significantly lower than the control group (*U* = 65, *Z* = -2.018, *P* = 0.044) (See Fig. 2C and D).

#### Time Spent in Target Quadrant (N = 15, n = 1)

The MWM tests were performed on 15 mice per group after anesthesia. On Day 5, Shapiro-Wilk normality test results for the experimental group were non-significant (*W* = 0.939, *P* = 0.374), indicating normally distributed data. Shapiro-Wilk test results for the control group were significant (*W* = 0.940, *P* = 0.382), indicating that the data were normally distributed. Levene’s test for homogeneity of variances on Day 5 scores showed a trend towards significance but did not reach the conventional threshold (*F*(1, 28) = 4.209, *P* = 0.05). Given this ambiguity and to be conservative, we used the Welch’s t-test which does not assume equal variances. The data for the target quadrant time percentage were normally distributed but exhibited heterogeneous variances, so we used Welch’s t-test to compare the differences between the two groups. The independent samples t-test (not assuming equal variances) showed no significant difference in the target quadrant time percentage between the two groups, *t*(21.89) = -1.007, *P* = 0.325, mean difference = -0.042, 95% CI [-0.128, 0.044], Cohen’s d = -0.368.

### Transcriptomics

#### Inter-Group Difference Analysis

**Fig. 3:**
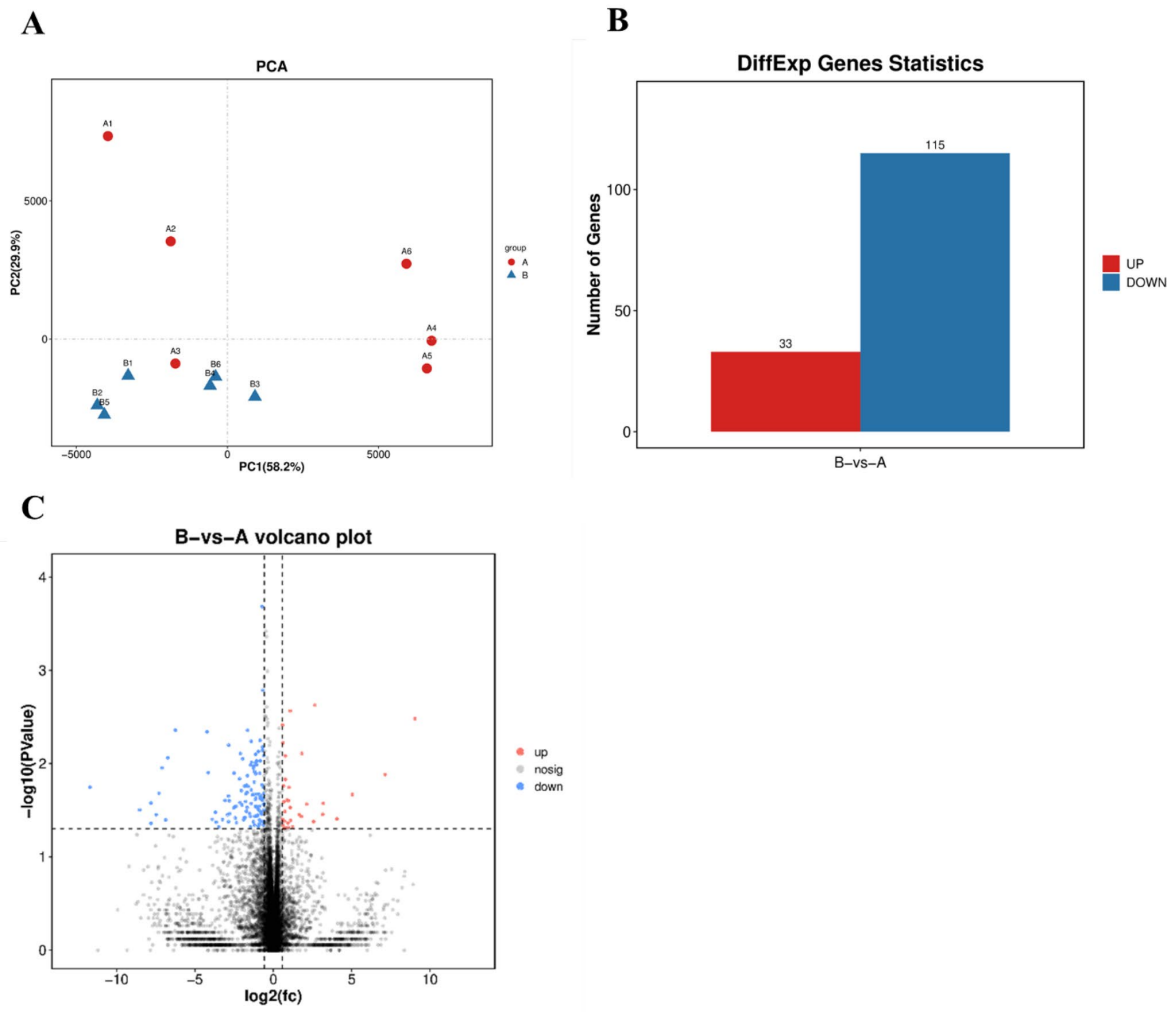
Inter-group Difference Analysis (*N* = 6). **A**: Principal Component Analysis; **B**: Bar Chart of Differentially Expressed Genes; **C**: Volcano Plot of Differentially Expresse. Letters ‘**A**’ and ‘**B**’ in the figure denote the experimental and control groups, respectively. Significantly differentially expressed genes (DEGs) were identified using a threshold of *P* < 0.05 and FC > 1.5. No genes passed the stringent threshold of FDR < 0.05. These plots are presented for exploratory purposes to visualize potential expression changes

As shown in Fig. 3A, principal component analysis (PCA) revealed that the first principal component (PC1) accounted for 58.7% of the intergroup variation and the second principal component (PC2) accounted for 29.9%. Cumulatively, these two components explained 88.1% of the variance. The sample groups demonstrated high reproducibility with no significant outliers detected.

When a stringent FDR correction (Q < 0.05) was applied, no statistically significant DEGs were identified. However, at an exploratory nominal p-value threshold (*P* < 0.05) and |log2FC| > log2(1.5), we observed 148 genes (33 upregulated, 115 downregulated) showing trends of differential expression, which were used for subsequent hypothesis-generating pathway analysis. As demonstrated in Fig. 3B, the number of upregulated genes is represented in red, and the number of downregulated genes is depicted in blue. Compared to the control group, the experimental group exhibited 33 upregulated genes and 115 downregulated genes. Figure 3C displays a volcano plot where the x-axis represents the log2-transformed fold change (FC) values and the y-axis shows the -log10-transformed *P*-values. Key thresholds are marked by dashed lines: A vertical dashed line on the y-axis corresponds to the significance cutoff (*P* = 0.05). Vertical dashed lines on the x-axis indicate the log2-transformed fold change thresholds (FC = 1.5,corresponding to log2(1.5) and log2(1/1.5)). Blue and red points represent genes with *P* < 0.05 and FC > 1.5. The analysis confirmed 115 downregulated genes and 33 upregulated genes.

#### Enrichment Analysis of Key Signaling Pathways

KEGG and GO pathway enrichment analysis of the candidate genes revealed one pathway that was significantly enriched after multiple-testing correction (FDR < 0.05): cysteine and methionine metabolism (FDR = 0.006). The complete results of the enrichment analysis are provided in Supplementary_Table_S1_Full_KEGG_Enrichment_Results and Supplementary_Table_S1_Full_GO_Enrichment_Results.

**Fig. 4:**
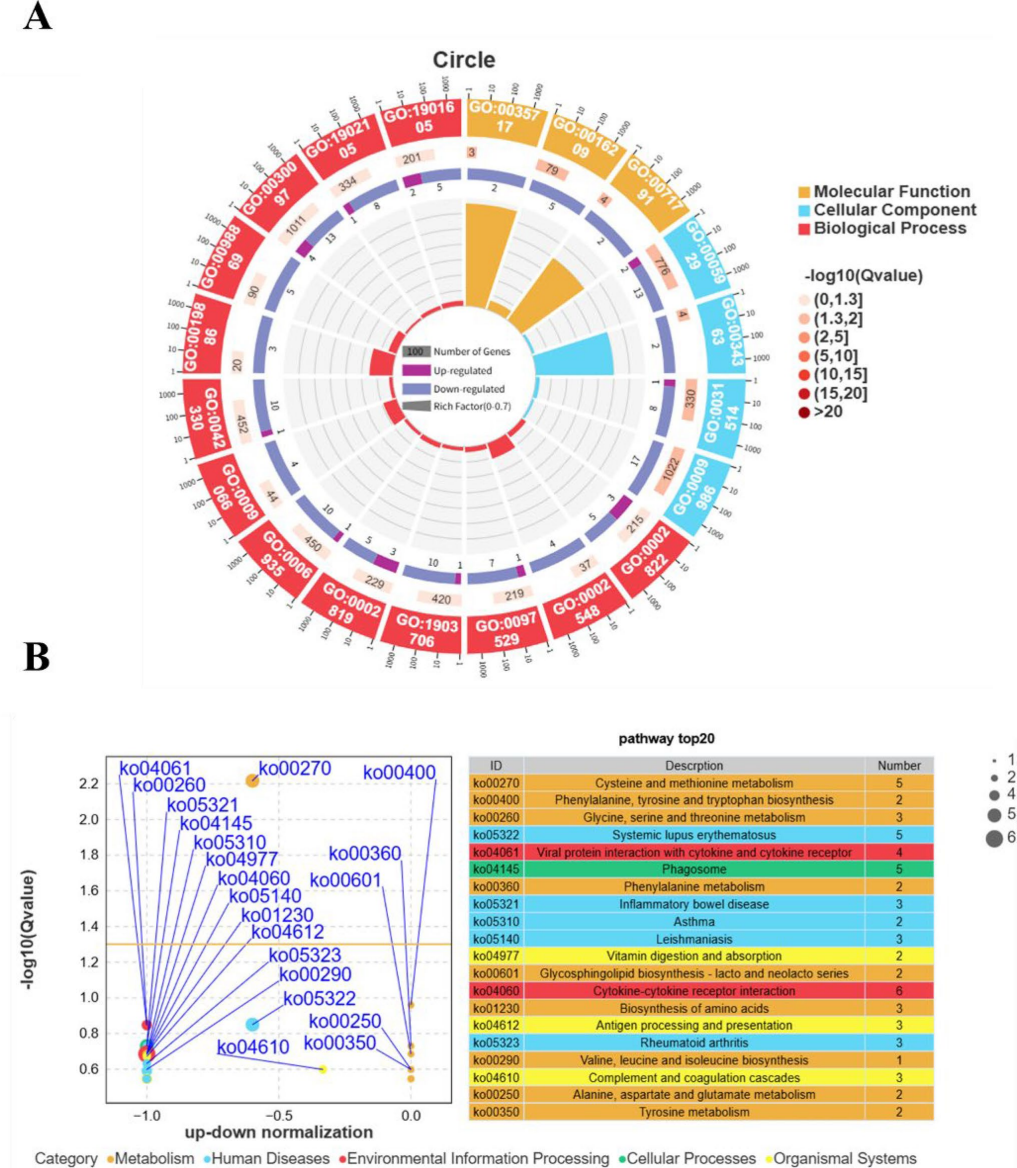
Enrichment Analysis of Key Signaling Pathways (*N* = 6, FDR < 0.05). **A**: Circular Diagram of Differential GO Enrichment; **B**: Bubble Plot of Differential KEGG Enrichment

As demonstrated in Fig. 4A, the outermost circle displays the number of DEGs associated with each GO term. The first inner circle lists the Top 20 enriched GO term IDs, with distinct colors representing different ontologies (Biological Process, Molecular Function, Cellular Component). The second circle illustrates the number of background genes enriched in each GO term (bar length) and their corresponding Q-values (color intensity: darker red indicates smaller Q-values). The third circle shows the proportion of upregulated (deep purple) and downregulated (light purple) DEGs within each GO term, with specific numerical values displayed below.The fourth (innermost) circle represents the ratio of DEGs enriched in a GO term to background genes enriched in the same GO term. Among the top 20 enriched GO terms, the top-ranked term in Biological Process was regulation of adaptive immune response based on somatic recombination of immune receptors built from immunoglobulin superfamily domains (GO:0002822). In Cellular Component, the highest-ranking term was cilium (GO:0005929), while the most significant term in Molecular Function was chemokine (C-C motif) ligand 7 binding (GO:0035717).

As shown in Fig. 4B, the x-axis represents the proportion of the difference between the number of upregulated and downregulated genes relative to the total DEGs. The y-axis displays the -log10-transformed *Q*-values for each pathway, where higher values indicate greater statistical significance. Each bubble corresponds to a pathway, with colors distinguishing different functional classifications (metabolism, immune response) and bubble size reflecting the total number of DEGs associated with the pathway. The right-side table lists the Top 20 enriched pathways, with the most significant pathway identified as KEGG ID: ko00270. According to the enrichment results, this ID corresponds to Cysteine and methionine metabolism. KEGG pathway enrichment analysis of the DEGs revealed that the most significantly enriched pathways included Cysteine and methionine metabolism. Furthermore, the analysis also suggested that the Platelet activation pathway might be affected (*P* = 0.220, *Q* = 0.532, enrichment score = 2.270), although its enrichment significance did not reach the level of the most prominent pathways. Detailed data can be found in the supplementary materials (Supplementary_Table_S1_Full_KEGG_Enrichment_Results). The figure below displays the significance pathway map of cysteine and methionine metabolism.

#### Cysteine and Methionine Metabolism

In this pathway, both upregulated and downregulated genes were observed. Among the significantly altered genes, the expression of the immunomodulatory enzyme Interleukin-4-Induced-1 (*IL4I1*) was significantly elevated. Conversely, L-serine/threonine ammonia-lyases (such as *SDS*, *SDH* or *CHA1*), cytoplasmic aspartate aminotransferase (*GOT1*), glycine N-methyltransferase (*GNMT*) and betaine-homocysteine S-methyltransferase (*BHMT*) were all significantly downregulated.

## Discussion

### Morris Water Maze Experiment

The screening MWM test revealed no differences between the two groups of aged mice prior to anesthesia, excluding the influence of “non-cognitive” factors (vision, thermoregulation, sensorimotor abilities, stress responses and anxiety-related behaviors) and pre-existing cognitive impairments in aged mice. Previous studies emphasize the necessity of screening out aged mice with “non-cognitive” factors (Kapadia et al. 2016; Olesen et al. 2024; Marcozzi et al. 2025), yet specific protocols for selecting eligible mice remain underexplored. In this study, a pilot MWM experiment was conducted analogous to the control group, demonstrating that mice gradually reduced their escape latency over time. This suggests that hippocampal-dependent memory formation improved with repeated training. Post-anesthesia MWM experiments evaluated the effects of sevoflurane exposure on cognitive function in aged mice. Compared to the control group, the sevoflurane-exposed group exhibited prolonged escape latency, increased path length, and reduced time spent in the target quadrant. These cognitive deficits align with prior findings (Zhang et al. 2022, 2025). Sevoflurane exposure impaired hippocampal neurofunction in aged mice, leading to learning deficits and deviations in spatial memory for platform location.

This study utilized rodents (mice) for MWM behavioral testing, leveraging their innate aversion to water to establish an experimental model. During the early learning phase of MWM, improved learning efficiency and shortened path lengths reflect the gradual optimization of navigational abilities toward the target location. As proficiency increased, spatially cued navigation strategies became dominant, while non-spatial orientation behaviors and repetitive circling decreased (Laeremans et al. 2015; García et al. 2025). Poor performance in initial trials may be attributed to sensorimotor alterations, where frequent thigmotaxis (wall-hugging behavior) significantly reduced swimming speed and increased floating time (Kapadia et al. 2016). Prolonged escape latency in experimental mice is more likely associated with non-cognitive factors such as motor dysfunction, thigmotaxis or increased floating time.

Previous research also indicated no differences in swimming speed between anesthesia-exposed and control groups (Zhou et al. 2024). Our experimental data contradict previous findings, as we observed a significant increase in swimming speed in sevoflurane-exposed mice on days 3 and 5 post-anesthesia, despite their prolonged escape latency. This finding argues against simple motor deficits being the primary cause of the extended escape delay and may also indirectly suggest that pure motor impairment is not a significant contributor to cognitive dysfunction. The presence of increased speed suggests that the performance deficit is more likely rooted in navigational strategy (Dembrow and Johnston 2014; Pfeiffer 2022) or anxiety domains(Yang et al. 2025; Musillo et al. 2025) rather than in general motor incapacity. Therefore, we propose that the hyperlocomotion likely reflects a strategy-based cognitive impairment (Parrini et al. 2024). The increased speed may be indicative of anxious hyperactivity or a compensatory yet inefficient and disorganized search strategy (Li et al. 2022). In such a scenario, animals unable to form or recall a spatial map (Tanaka et al. 2018; Varga et al. 2025), resort to frantic, non-cognitive swimming patterns. The faster swimming could represent a less focused, thigmotactic (wall-hugging), or frantic search pattern (García et al. 2025), which is inefficient and ultimately results in longer latencies to find the platform.

The results revealed a significant negative correlation between center time and escape latency, indicating that mice spending more time exploring the center exhibited better learning performance (Table 1; Fig. 2A and B). We have now provided representative swimming paths for both groups during both the place navigation task and the spatial probe test to visually illustrate differences in search strategies (Fig. 2). The comprehensive behavioral profile suggests that the primary deficit in the experimental group lies in spatial learning and memory, although alternative explanations cannot be entirely ruled out: the experimental group showed significantly longer escape latencies during the place navigation task and a significant reduction in platform crossings during the spatial probe test. Contrary to our initial expectations, the time spent in the target quadrant by the experimental group, while lower than the control group, did not reach statistical significance. This indicates that while memory retrieval might be impaired, the effect on this specific metric is less pronounced. The observed reduction in center time and the higher swimming velocity are consistent with potential anxiety-like behavior or hyperlocomotion. However, the most robust findings remain centered on the learning and memory deficits. We fully agree that distinguishing between anxiety, motor function and cognitive strategy requires more specialized experiments. Therefore, we have explicitly added a statement to the Future Perspectives section of the manuscript outlining plans to address this in subsequent studies. The increased speed is not an indicator of superior cognition; rather, it likely stems from anxiety-like behavior or a dysfunctional search strategy, which causes the animals to move more rapidly but with compromised efficiency. Thus, the paradoxical combination of increased speed and prolonged latency strengthens our conclusion that sevoflurane anesthesia primarily disrupts spatial learning and executive function, rather than general motor capacity (Chen et al. 2022a, b; Tang et al. 2025; Li et al. 2025).

Chao et al. (Chao et al. 2022) propose that the the rodent hippocampus is involved in Novel Object Recognition (NOR) task and memory encoding, consolidation and retrieval-based on familiarity or recall-depend on hippocampal integrity. The hippocampal formation is the brain region that regulates cognitive and emotional processes, primarily serving learning, memory and cognitive functions (Chang et al. 2021; Yin et al. 2024). The functional organization of hippocampal excitatory synapses critically impacts cognitive performance. Long-term cognitive changes induced by anesthetic exposure are mechanistically linked to altered surface expression of extrasynaptic GABAA receptors (Wang et al. 2024a). Volatile anesthetics exert neuroregulatory effects by targeting synaptic and extrasynaptic GABAA receptors, binding specifically to the transmembrane domains of α1β3γ2LGABAA receptors (Woll et al. 2018). Four hours of sevoflurane anesthesia suppressed light-induced Per2 mRNA expression in the suprachiasmatic nucleus, resulting in delayed circadian activity rhythms (Vacas et al. 2013). In neurodegenerative research, genetic mutations, protein-protein interactions, aberrant protein propagation mechanisms and dysregulation of synthesis-degradation homeostasis negatively influence the aggregation propensity of α-synuclein, contributing to cognitive decline in Parkinson’s disease patients (Fan et al. 2021). While impaired memory formation or retrieval cannot be entirely excluded, prolonged escape latency in experimental mice is more likely attributable to non-cognitive factors such as motor deficits (reduced swimming speed), thigmotaxis or increased floating time. Previous studies minimized auditory, visual and olfactory inputs while utilizing visual cues to assess navigational efficiency, effectively distinguishing cognitive deficits from motor or emotion-related behavioral changes (Kapadia et al. 2016; Colmant et al. 2025; Zhao et al. 2025a).

### Transcriptomics

It is important to note that the transcriptomic changes observed did not reach statistical significance (FDR < 0.05) after rigorous multiple testing correction. Therefore, the following discussion regarding pathway enrichment is exploratory and hypothesis-generating in nature. The implicated associations require future validation and do not establish causation. Transcriptomic analysis in this study identified 148 DEGs between the experimental and control groups, with 33 upregulated and 115 downregulated genes. Regarding the direction of differential gene expression relative to baseline, downregulated genes exhibited absolute predominance. This suggests that sevoflurane anesthesia is associated with a widespread suppression of gene expression or deactivation of functional networks in the brains of aged mice.

With the above cautionary note in mind, we proceeded to examine the top-enriched pathway and its constituent genes at a nominal significance threshold (*P* < 0.05, |log2FC| > log2(1.5)). The most prominent finding was the enrichment of the cysteine and methionine metabolism pathway. While statistically provisional, the coordinated changes in the following key enzymes within this pathway suggest a potential metabolic axis worthy of further investigation:

In the pathway map, after adjusting for multiple comparisons using the FDR, the cysteine and methionine metabolism pathway remained statistically significant (FDR < 0.05). Within this pathway, the expression of the significantly altered immunomodulatory enzyme *IL4I1* was markedly elevated. Previous studies have established *IL4I1* as a key target in tumor immunomodulation, which operates by depleting tryptophan to activate the aryl hydrocarbon receptor signaling pathway (Zhao et al. 2025b). According to previous studies, this promotes the formation of an immune-tolerant microenvironment and exacerbates inflammatory responses (Shen et al. 2024). Chronic inflammation is a core mechanism underlying cognitive impairment in conditions such as Alzheimer’s disease (Kinney et al. 2018). Consistent with an exploratory hypothesis, the upregulation of *IL4I1*, a gene known to be involved in immunomodulation, hints at a possible link between sevoflurane exposure and processes related to neuroinflammation, which has been implicated in cognitive dysfunction.

Transcriptomic data revealed downregulation of L-serine/threonine ammonia-lyases (*SDS*, *SDH*, or homologous enzymes), suggesting a potential association between sevoflurane exposure and disruption of the amino acid metabolic network in the aged brain.Impaired conversion of the metabolic hub serine (Baraniuk 2025) and inhibition of cysteine metabolism (*GCLC/GCLM*) were observed. This pattern of gene expression could be consistent with a constrained capacity for glutathione synthesis, which might, in turn, be associated with weakened antioxidant defenses. The overall metabolic profile shares features with models of oxidative stress, though a direct role for ferroptosis remains speculative and was not tested here(Venkateshappa et al. 2012).

Concurrently, potential reductions in D-serine generation (Garofalo et al. 2024) impair N-methyl-D-aspartate receptor-mediated synaptic plasticity (Coyle et al. 2020; Errico et al. 2020; Souza et al. 2023). Obstruction of threonine conversion to acetyl-CoA is expected to reduce mitochondrial tricarboxylic acid (TCA) cycle flux. As the TCA cycle is a core component of energy metabolism (Jiao et al. 2024), this may exacerbate the inherent mitochondrial energy crisis in aged neurons (Mahapatra et al. 2024), potentially contributing to cognitive impairment in the elderly. By inhibiting these hub metabolic enzymes, sevoflurane likely depletes the already diminished antioxidant and energy reserves of the aged brain. This could amplify the metabolic fragility of aging, possibly driving cognitive decline through oxidative damage and synaptic dysfunction. The functional integration of these enzyme effects is consistent with and strengthens the “metabolic reprogramming drives cognitive impairment” theory, providing novel mechanistic insight into anesthesia-induced cognitive decline.

Our results indicate that the downregulation of *GOT1* may be linked to sevoflurane-induced alterations in energy metabolism and redox homeostasis in the aged brain. *GOT1* catalyzes the conversion of aspartate and α-ketoglutarate to oxaloacetate and glutamate. We therefore hypothesize that reduced oxaloacetate generation may impair TCA cycle flux, while impaired malate-aspartate shuttle activity could lead to cytosolic NADH accumulation. It is plausible that these potential alterations, when combined with age-associated mitochondrial dysfunction (Goleij et al. 2025; Santana et al. 2025), could exacerbate ATP deficits in aged neurons—overlapping with mechanisms described earlier. Concurrently, a potential constrained glutamate supply (a key glutathione precursor) may synergize with downregulated *GCLC/GCLM*, which could lead to a systemic decline in free radical scavenging capacity (Xia et al. 2015; Peng et al. 2015; Wang et al. 2016). The downregulation of *GOT1* suggests a potential alteration in the malate-aspartate shuttle and TCA cycle anaplerosis. It is plausible that these transcriptional changes, if reflected at the functional level, could exacerbate the energy deficits common in the aged brain. We speculate that this might form part of a self-perpetuating cycle contributing to metabolic stress (Dai et al. 2024; de Macedo et al. 2025).

These data suggest that *GNMT* downregulation may contribute to profound disruption of one-carbon metabolism by sevoflurane. As a key regulator of methylation cycling, *GNMT* deficiency is predicted to lead to SAM accumulation, potentially driving aberrant DNA/histone hypermethylation that could suppress synaptic plasticity gene transcription—suggesting a potential mechanism of epigenetic dysregulation (Liu et al. 2025). SAM might inhibit respiratory chain Complex IV, which could exacerbate inherent energy crises and oxidative stress in aged neurons (Y et al. 2025). Reduced S-adenosylhomocysteine may indirectly elevate homocysteinemia (Hcy), thereby potentially amplifying neurodegeneration via excitotoxicity and vascular injury (Yang et al. 2024c; Emrich et al. 2025). If validated, the downregulation of *GNMT* would point to a potential disruption in one-carbon metabolism. This could theoretically influence epigenetic regulation and homocysteine levels, both of which have been independently associated with cognitive decline. Thus, under our exploratory framework, *GNMT* represents a candidate node linking metabolic and potential epigenetic alterations in this context. Thus, *GNMT*—a critical node at the metabolism-epigenetics interface—could represent a core mechanism in sevoflurane-induced cognitive decline in the aged brain, positioning it as a novel candidate for preventive targets against anesthesia-related cognitive impairment.

Transcriptomic data show significant downregulation of *BHMT* mRNA in the hippocampus of aged mice post-sevoflurane. Based on its known biological role, this downregulation suggests a potential impairment in homocysteine clearance, which might lead to Hcy accumulation. Hyperhomocysteinemia has been linked to pathological cascades that include oxidative stress, neuroinflammation, neuronal damage/apoptosis, and impaired synaptic plasticity (Decaix et al. 2025), mechanisms that may ultimately manifest as neurocognitive dysfunction. This suggests an association between sevoflurane and altered homocysteine metabolism, which is a known risk factor for cognitive impairment, though a causal link here is not established. Collectively, these findings position *BHMT* as a potential critical nexus connecting anesthetic stress, aging, metabolic dysregulation, and neural injury alongside cognitive impairment. Furthermore, the aged brain exhibits baseline vulnerabilities such as enhanced neuroinflammation, diminished synaptic plasticity reserves, mitochondrial dysfunction, weakened antioxidant capacity, increased blood-brain barrier permeability and impaired toxin clearance efficiency.

As detailed above, we have elaborated on how significantly altered molecules within the cysteine/methionine pathway may impact cognitive function. Dysregulation of the cysteine/methionine metabolic pathway may activate glial cells by altering redox balance (Peña 2025) and methylation status (Maejima et al. 2025), leading to the release of pro-inflammatory cytokines and thereby exacerbating neuroinflammation. The integrated effects of oxidative stress, epigenetic changes, and neuroinflammation are likely to create a toxic environment for neurons. These alterations may collectively form a self-perpetuating cycle of metabolic-oxidative stress that significantly compromises the antioxidant capacity, energy supply, and synaptic function of the aging brain. Ultimately, cognitive decline may occur through multiple mechanisms, including oxidative damage, inflammatory responses, epigenetic silencing, and mitochondrial dysfunction. These findings not only reinforce the theory that “metabolic reprogramming drives cognitive impairment” (Qiang et al. 2021; Wu et al. 2025) but also provide novel mechanistic insights and potential therapeutic targets for anesthesia-related cognitive decline.

Our transcriptomic profiling provides a preliminary, systems-level view of hippocampal gene expression alterations following sevoflurane exposure in aged mice. The central, though statistically unvalidated, theme points toward dysregulation of the cysteine and methionine metabolism network. It is imperative to reiterate that the functional consequences of these transcriptional trends and their direct causal role in the observed cognitive deficit remain unknown and are fertile ground for future targeted experimentation. Beyond this primary pathway, our analysis yielded an additional, albeit less significant, observation that merits mention in a hypothesis-generating context.

Our transcriptomic profiling provides a preliminary, systems-level view of hippocampal gene expression alterations following sevoflurane exposure in aged mice. Through transcriptomic analysis, this study reveals disturbances in the cysteine and methionine metabolic network, which may represent a key molecular mechanism underlying neurocognitive disorders. It is imperative to reiterate that the functional consequences of these transcriptional trends and their direct causal role in the observed cognitive deficit remain unknown and are fertile ground for future targeted experimentation. Furthermore, our analysis provides an unexpected clue. Although the enrichment significance was limited, the identification of the platelet activation pathway is thought-provoking. Platelets are not merely coagulation components but can also amplify inflammatory responses by releasing inflammatory factors and directly interacting with immune cells (Yeung et al. 2018; Wagenhäuser et al. 2024). The enrichment of the platelet activation pathway, while preliminary, suggests an intriguing association that warrants further investigation. Platelets are key mediators of chronic inflammation and prothrombotic states (Müller et al. 2015), and some studies have confirmed that abnormal platelet activity may be associated with cognitive impairment (Gallo et al. 2024). Antiplatelet agents targeting these pathways, traditionally used in cardiovascular diseases, require validation through extensive clinical trials for their role in cognitive protection. However, whether platelet activation markers can serve as biomarkers for cognitive impairment remains questionable, and it is necessary to distinguish between causality (primary etiology) and correlation (accompanying phenomenon). It must be emphasized that this association is currently based solely on bioinformatic predictions and requires direct validation through future targeted experiments (e.g., measuring platelet activity markers, conducting in vitro intervention assays). Platelets are emerging as players in neuro-inflammation, and this finding opens a new hypothesis regarding their role in anesthesia-related cognitive changes, which should be tested in future studies.

When the neuro-immune-endocrine network is exposed to chronic stress, structural damage across organs, including the brain, exacerbates functional behavioral deficits (Kapadia and Sakic 2011; Picard et al. 2021). Under pathological conditions, exposure to pathogens or endogenous stressors (tumors, inflammation, auto-reactive cells, free radicals, amyloid proteins) (Li et al. 2017; Hamilton et al. 2024), triggers compensatory adaptive mechanisms through multisystem coordination to maintain homeostasis. Excessive inflammatory activation disrupts synaptic plasticity, induces neurotransmitter imbalances, and impairs interneuronal signaling, leading to cognitive dysfunction (Han et al. 2021). Inflammatory mediators also compromise blood-brain barrier integrity (Candelario-Jalil et al. 2022), allowing peripheral cytokines to infiltrate the central nervous system and establish a neuroinflammatory-neurodegenerative feedback loop, accelerating cognitive decline. Under pathological conditions, organisms initiate compensatory adaptive mechanisms through dynamic coordination across multiple systems to maintain homeostasis and promote survival. This compensatory response involves intricate regulation at molecular, cellular, and systemic levels, with its underlying principle focused on optimizing resource allocation through energy reallocation and threat prioritization.

The coordinated dysregulation of the cysteine and methionine metabolism pathway may represent a key mechanism underlying hippocampal vulnerability in aged mice following sevoflurane exposure. The hippocampus, crucial for learning and memory, is particularly reliant on robust antioxidant defense (Li et al. 2023), efficient energy metabolism (Brivio et al. 2022; Dong et al. 2023) and dynamic epigenetic regulation (Sanacora et al. 2022)—all of which are processes supported by this pathway. Our findings suggest that sevoflurane inflicts a “multi-hit” insult on the aged hippocampus. The downregulation of genes like GCLC/GCLM and the disruption of the methionine cycle (via GNMT and BHMT) likely impair glutathione synthesis and elevate homocysteine, compromising antioxidant capacity and promoting a pro-oxidant environment. This is particularly detrimental to the metabolically active and oxidatively sensitive hippocampal neurons. The downregulation of GOT1 and enzymes involved in serine/threonine catabolism may constrain TCA cycle flux and ATP generation, exacerbating the inherent energy deficit in the aging brain and failing to meet the high metabolic demands of synaptic plasticity. The potential for SAM accumulation due to GNMT downregulation threatens to cause aberrant hypermethylation, which could epigenetically silence genes critical for hippocampal synaptic function and memory formation. Thus, the cysteine and methionine metabolism pathway sits at a critical nexus, and its dysregulation by sevoflurane concurrently disrupts redox homeostasis, energy production, and epigenetic fidelity, thereby rendering the aged hippocampus highly susceptible to functional decline and manifesting as the observed cognitive deficits.

### Limitations

While this study provides initial insights, several important limitations must be acknowledged to properly contextualize the findings and guide future research.

The primary limitation lies in the exploratory nature and validation gap of the transcriptomic data. The foremost limitation is the exploratory nature of the transcriptomic data. As noted, no differentially expressed genes (DEGs) survived strict genome-wide multiple testing correction (FDR < 0.05). The transcriptomic findings, particularly the alterations in the cysteine and methionine metabolism pathway and the individual genes discussed (e.g., IL4I1, SDS, GOT1, GNMT, BHMT), are based solely on RNA sequencing data and lack validation by independent experimental methods such as qPCR, western blot, or functional assays. Consequently, the observed gene expression changes and their potential biological implications remain associative and require further confirmation. The sample size for the transcriptomic analysis (*n* = 6 per group), while consistent with many initial RNA-seq studies, is relatively small. The sample size affords limited statistical power to detect subtle but biologically important changes and increases the risk of both false positives and false negatives. This limited statistical power is reflected in the fact that no genes passed a stringent FDR correction, and it increases the risk of both Type I and Type II errors. Consequently, the identified pathway and genes represent promising leads, not established mechanisms. A larger sample size would be necessary to enhance the robustness and generalizability of the transcriptomic signatures identified. More fundamentally, this study stops at the level of mRNA expression, which presents a significant inferential gap. Changes in transcript abundance do not necessarily equate to changes in protein levels, enzymatic activity, or metabolic flux. Without proteomic, metabolomic, or functional biochemical data, the actual metabolic consequences of sevoflurane exposure remain speculative. Future work must bridge this gap to confirm whether the observed transcriptional shifts translate to functional pathway disruption.

Second, the limitations of the research model and design affect the generalizability of the conclusions. Our study was conducted exclusively in male mice, which limits the generalizability of our findings to female populations. This is an important constraint, as substantial evidence indicates the existence of sex differences in cognitive impairment processes (Srs et al. 2025; Baghdadchi et al. 2025; Petrič et al. 2025; Olejnik et al. 2025). Consequently, our present conclusions are necessarily confined to the male sex. Furthermore, the model does not incorporate common age-related co-morbidities (e.g., vascular disease, metabolic syndrome) that likely interact with anesthesia to exacerbate cognitive decline in human patients. The generalizability of our findings to the heterogeneous elderly surgical population is inherently limited. Our use of a healthy, aged, and male mouse model simplifies a complex clinical reality. Additionally, all transcriptomic data were derived from hippocampal tissue, leaving open the question of whether similar associations exist in other brain regions relevant to cognition. The hippocampus is a heterogeneous structure composed of distinct subregions and diverse cell types. The observed transcriptomic signature represents an average across all these elements. Single-cell or spatial transcriptomics in future studies will be essential to deconvolve this complexity and assign molecular changes to specific cellular actors.

Third, there are inherent boundaries in the behavioral assessment and statistical methods. The MWM test, while standard, cannot definitively distinguish between specific types of memory deficits and complementary behavioral assays would provide a more nuanced phenotypic analysis. The observed deficits could arise from impairments in acquisition, consolidation, retrieval, or a combination thereof. This study utilized the MWM test, which cannot distinguish short-term working memory from long-term episodic memory deficits. Complementary assays such as NOR task (Reger et al. 2009; Da Cruz et al. 2020) are needed to refine behavioral phenotyping. This limits the precision with which we can link specific transcriptomic changes to specific behavioral domains. Furhermore, in the statistical modeling of behavioral data, only random intercepts were included due to sample size constraints; the inclusion of random slopes could provide a more comprehensive understanding of individual trajectories over time. Random slopes were not tested due to sample size limitations, which could affect the generalizability of the findings across all time points.

Finally, this study establishes an association rather than a causal relationship. This is a correlative study. We demonstrate an association between sevoflurane exposure, behavioral deficits, and hippocampal transcriptomic alterations. Definitive proof of causality requires interventional studies, such as pharmacological or genetic manipulation of key nodes (e.g., GNMT, BHMT) to see if they can rescue or exacerbate the sevoflurane-induced phenotype. Similarly, this study suggests that the platelet activation pathway may be linked to sevoflurane-induced cognitive impairment, but its mechanism requires validation through integrated in vivo and in vitro models. The conclusions are limited in generalizability due to reliance on animal experiments without clinical sample data. The potential therapeutic value of antiplatelet interventions needs further experimental confirmation, though the platelet activation pathway could emerge as a novel target for cognitive impairment prevention.

### Future Directions

The underlying motivation for the observed increases in velocity and alterations in behavioral patterns in this study—such as anxiety, motor discoordination or alternative search strategies—remains to be fully determined. To address this issue directly in future research, we plan to incorporate specialized behavioral assays in subsequent animal cohorts. The Open Field test and Elevated Plus Maze will be used to quantify anxiety-like behavior. Gait analysis will be employed to assess fundamental motor coordination and control. Strategy analysis will be conducted in more complex mazes (such as the Barnes maze or the 8-arm radial maze) to dissect cognitive search strategies more finely. Our team is currently planning a follow-up study designed to directly replicate the experiments performed here using age-matched female mice. This will enable a direct comparison of the core findings between sexes. Data from male and female cohorts will be analyzed both separately and pooled with sex as a biological variable to identify any significant interactions. We believe this plan will not only corroborate our current findings in males but will also significantly advance the field by providing a comprehensive understanding of their expression across sexes. We have identified key genes, but targeted research on them is still lacking. Our team may subsequently employ knockout models or pharmacological rescue experiments targeting these critical genes to further establish a causal relationship with sevoflurane-induced cognitive impairment. For the key genes *GNMT*, *SDS*,* SDH*,* GOT1*,* GNMT*,* BHMT*, and *GOT1*, we will perform targeted loss-of-function (LoF) and gain-of-function (GoF) experiments using genetic tools in neuronal models. This study highlights the novel role of platelet activation in cognitive impairment but lacks mechanistic validation. Future research should employ platelet inhibitors to test their efficacy in mitigating sevoflurane-induced cognitive deficits. Clinically, these findings urge anesthesiologists to critically evaluate sevoflurane’s neurocognitive risks and inform safer protocols. Mechanistic insights into sevoflurane’s effects on cognition may guide the development of targeted therapies, bridging preclinical discoveries to clinical applications.

## Conclusions

This exploratory transcriptomic analysis suggests a potential association between sevoflurane anesthesia and coordinated transcriptional alterations in the cysteine and methionine metabolic network in the hippocampus of aged mice. The gene expression patterns, while requiring confirmation, are consistent with a model where perturbations in antioxidant defense, energy metabolism, and neuro-immune signaling may collectively contribute to cognitive impairment. The gene expression patterns are consistent with coordinated changes in several biological processes, including antioxidant defense, energy metabolism, and neuro-immune signaling, which may collectively contribute to a metabolic-stress model of cognitive impairment. Furthermore, the enrichment of the platelet activation pathway hints at a potential novel axis of neuro-immune-coagulation crosstalk. Together, these processes drive a self-perpetuating metabolic-oxidative stress cycle. Furthermore, enrichment of the platelet activation pathway suggests a potential role of neuro-immune-coagulation crosstalk in cognitive impairment. These findings strongly support the theoretical framework that “metabolic reprogramming drives cognitive impairment” and provide novel mechanistic insights into anesthesia-induced neurocognitive decline, highlighting potential therapeutic targets.

## Supplementary Information

Below is the link to the electronic supplementary material.

Supplementary Material 1

Supplementary Material 2

Supplementary Material 3

Supplementary Material 4

Supplementary Material 5

## Data Availability

The data discovered in this study have been deposited in the China National GeneBank Database (CNGBdb) of the China National GeneBank Sequence Archive (CNSA), with the accession number CNP0007894.

## References

[CR1] Baghdadchi Y, White C, Dimitrov E (2025) Obesity suppresses hippocampal neurogenesis and impairs memory and executive function in a weight and sex-specific manner. Physiol Behav. 10.1016/j.physbeh.2025.115141 10.1016/j.physbeh.2025.115141 PMC13571213 41130433

[CR2] Baraniuk JN (2025) Cerebrospinal fluid metabolomics, lipidomics and Serine pathway dysfunction in myalgic encephalomyelitis/chronic fatigue syndroome (ME/CFS). Sci Rep 15:7381. 10.1038/s41598-025-91324-1 40025157 10.1038/s41598-025-91324-1 PMC11873053

[CR3] Berian JR, Zhou L, Russell MM et al (2018) Postoperative delirium as a target for surgical quality improvement. Ann Surg 268:93–99. 10.1097/SLA.0000000000002436 28742701 10.1097/SLA.0000000000002436

[CR4] Brivio P, Audano M, Gallo MT et al (2022) Metabolomic signature and mitochondrial dynamics outline the difference between vulnerability and resilience to chronic stress. Transl Psychiatry 12:87. 10.1038/s41398-022-01856-7 35228511 10.1038/s41398-022-01856-7 PMC8885712

[CR5] Candelario-Jalil E, Dijkhuizen RM, Magnus T (2022) Neuroinflammation, stroke, blood-brain barrier dysfunction, and imaging modalities. Stroke 53:1473–1486. 10.1161/STROKEAHA.122.036946 35387495 10.1161/STROKEAHA.122.036946 PMC9038693

[CR6] Chang H-M, Lin H-C, Cheng H-L et al (2021) Melatonin successfully rescues the hippocampal molecular machinery and enhances anti-oxidative activity following early-life sleep deprivation injury. Antioxid (basel Switz) 10:774. 10.3390/antiox10050774 10.3390/antiox10050774 PMC8153000 34068192

[CR7] Chao OY, Nikolaus S, Yang Y-M, Huston JP (2022) Neuronal circuitry for recognition memory of object and place in rodent models. Neurosci Biobehav Rev 141:104855. 10.1016/j.neubiorev.2022.104855 36089106 10.1016/j.neubiorev.2022.104855 PMC10542956

[CR9] Chen S, Zhou Y, Chen Y, Gu J (2018) Fastp: an ultra-fast all-in-one FASTQ preprocessor. Bioinform (Oxf Engl) 34:i884–i890. 10.1093/bioinformatics/bty560 10.1093/bioinformatics/bty560 PMC6129281 30423086

[CR8] Chen K, Hu Q, Gupta R et al (2022a) Inhibition of unfolded protein response prevents post-anesthesia neuronal hyperactivity and synapse loss in aged mice. Aging Cell 21:e13592. 10.1111/acel.13592 35299279 10.1111/acel.13592 PMC9009124

[CR10] Chen Y-R, Zhang S-X, Fang M et al (2022b) Egr2 contributes to age-dependent vulnerability to sevoflurane-induced cognitive deficits in mice. Acta Pharmacol Sin 43:2828–2840. 10.1038/s41401-022-00915-5 35577909 10.1038/s41401-022-00915-5 PMC9622904

[CR11] Colmant L, Quenon L, Huyghe L et al (2025) Rotation errors in path integration are associated with alzheimer’s disease Tau pathology: a cross-sectional study. Alzheimer’s Res Ther 17:34. 10.1186/s13195-025-01679-w 39893494 10.1186/s13195-025-01679-w PMC11786419

[CR12] Coyle JT, Balu D, Wolosker H (2020) D-serine, the shape-shifting NMDA receptor co-agonist. Neurochem Res 45:1344–1353. 10.1007/s11064-020-03014-1 32189130 10.1007/s11064-020-03014-1 PMC7313399

[CR13] Da Cruz JFO, Gomis-Gonzalez M, Maldonado R et al (2020) An alternative maze to assess novel object recognition in mice. Bio-protoc 10:e3651. 10.21769/BioProtoc.3651 33659321 10.21769/BioProtoc.3651 PMC7842337

[CR14] Dai C, Fu C, Yi Z et al (2024) Sirtuin 3 (SIRT3) improves sevoflurane-induced postoperative cognitive impairment by regulating mitochondrial oxidative stress. Cell Mol Biol (noisy-le-gd Fr) 70:62–66. 10.14715/cmb/2024.70.2.9 10.14715/cmb/2024.70.2.9 38430040

[CR15] de Macedo GT, Claro MT, Ferreira SA et al (2025) Mitochondrial and inflammatory dysfunctions underlie perfluorooctane sulfonic acid (PFOS)-induced neurotoxicity in adult zebrafish. Sci Total Environ 992:179972. 10.1016/j.scitotenv.2025.179972 40570396 10.1016/j.scitotenv.2025.179972

[CR16] Decaix T, Lilamand M, Götze K et al (2025) Unraveling homocysteine’s role in dementia: no specific association with alzheimer’s disease, but a connection to white matter hyperintensities. Aging Dis. 10.14336/AD.225.0020 40249931 10.14336/AD.225.0020 PMC12834409

[CR17] Dembrow N, Johnston D (2014) Subcircuit-specific neuromodulation in the prefrontal cortex. Front Neural Circuits 8:54. 10.3389/fncir.2014.00054 24926234 10.3389/fncir.2014.00054 PMC4046580

[CR18] Dong W-T, Long L-H, Deng Q et al (2023) Mitochondrial fission drives neuronal metabolic burden to promote stress susceptibility in male mice. Nat Metab 5:2220–2236. 10.1038/s42255-023-00924-6 37985735 10.1038/s42255-023-00924-6

[CR19] Emrich IE, Obeid R, Geisel J et al (2025) Association of homocysteine, S-adenosylhomocysteine and S-adenosylmethionine with cardiovascular events in chronic kidney disease. Nutrients 17:626. 10.3390/nu17040626 40004955 10.3390/nu17040626 PMC11858042

[CR20] Errico F, Cuomo M, Canu N et al (2020) New insights on the influence of free d-aspartate metabolism in the mammalian brain during prenatal and postnatal life. Biochim Biophys Acta Proteins Proteom 1868:140471. 10.1016/j.bbapap.2020.140471 32561430 10.1016/j.bbapap.2020.140471

[CR21] Fan T-S, Liu SC-H, Wu R-M (2021) Alpha-Synuclein and cognitive decline in Parkinson disease. Life (Basel) 11:1239. 10.3390/life11111239 34833115 10.3390/life11111239 PMC8625417

[CR22] Gallo A, Lipari A, Di Francesco S et al (2024) Platelets and neurodegenerative diseases: current knowledge and future perspectives. Int J Mol Sci 25:6292. 10.3390/ijms25126292 38927999 10.3390/ijms25126292 PMC11203688

[CR23] García F, Torres M-J, Chacana-Véliz L et al (2025) Prefrontal cortex synchronization with the hippocampus and parietal cortex is strategy-dependent during Spatial learning. Commun Biol 8:79. 10.1038/s42003-025-07486-1 39825081 10.1038/s42003-025-07486-1 PMC11742664

[CR24] Garofalo M, De Simone G, Motta Z et al (2024) Decreased free D-aspartate levels in the blood serum of patients with schizophrenia. Front Psychiatry 15:1408175. 10.3389/fpsyt.2024.1408175 39050919 10.3389/fpsyt.2024.1408175 PMC11266155

[CR25] Geng Y-J, Wu Q-H, Zhang R-Q (2017) Effect of propofol, sevoflurane, and isoflurane on postoperative cognitive dysfunction following laparoscopic cholecystectomy in elderly patients: a randomized controlled trial. J Clin Anesth 38:165–171. 10.1016/j.jclinane.2017.02.007 28372661 10.1016/j.jclinane.2017.02.007

[CR26] Goleij P, Khazeei Tabari MA, Poudineh M et al (2025) Therapeutic potential of melatonin-induced mitophagy in the pathogenesis of alzheimer’s disease. Inflammopharmacology 33:4553–4575. 10.1007/s10787-025-01859-y 40694204 10.1007/s10787-025-01859-y

[CR27] Hamilton OS, Iob E, Ajnakina O et al (2024) Immune-neuroendocrine patterning and response to stress. A latent profile analysis in the english longitudinal study of ageing. Brain Behav Immun 115:600–608. 10.1016/j.bbi.2023.11.012 37967661 10.1016/j.bbi.2023.11.012

[CR28] Han C, Zhang Z, Guo N et al (2021) Effects of Sevoflurane inhalation anesthesia on the intestinal microbiome in mice. Front Cell Infect Microbiol 11:633527. 10.3389/fcimb.2021.633527 33816336 10.3389/fcimb.2021.633527 PMC8012717

[CR81] Hy YS, Qq L Y, et al (2025) Unveiling moxibustion’s impact on AD mice learning and memory: role of mitochondrial respiratory chain complex I subunit in the hippocampus. Mol Neurobiol. 10.1007/s12035-025-05147-2 10.1007/s12035-025-05147-2 40531365

[CR29] Jiao J, Gao G, Zhu J et al (2024) Binding of α-synuclein to ACO2 promotes progressive mitochondrial dysfunction in parkinson’s disease models. Redox Biol 77:103399. 10.1016/j.redox.2024.103399 39427443 10.1016/j.redox.2024.103399 PMC11533713

[CR30] Kapadia M, Sakic B (2011) Autoimmune and inflammatory mechanisms of CNS damage. Prog Neurobiol 95:301–333. 10.1016/j.pneurobio.2011.08.008 21889967 10.1016/j.pneurobio.2011.08.008

[CR31] Kapadia M, Xu J, Sakic B (2016) The water maze paradigm in experimental studies of chronic cognitive disorders: theory, protocols, analysis, and inference. Neurosci Biobehav Rev 68:195–217. 10.1016/j.neubiorev.2016.05.016 27229758 10.1016/j.neubiorev.2016.05.016

[CR32] Kinney JW, Bemiller SM, Murtishaw AS et al (2018) Inflammation as a central mechanism in alzheimer’s disease. Alzheimer’s Dement (n Y N Y) 4:575–590. 10.1016/j.trci.2018.06.014 10.1016/j.trci.2018.06.014 PMC6214864 30406177

[CR33] Laeremans A, Sabanov V, Ahmed T et al (2015) Distinct and simultaneously active plasticity mechanisms in mouse hippocampus during different phases of Morris water maze training. Brain Struct Funct 220:1273–1290. 10.1007/s00429-014-0722-z 24562414 10.1007/s00429-014-0722-z

[CR34] Li J, Labbadia J, Morimoto RI (2017) Rethinking HSF1 in stress, development, and organismal health. Trends Cell Biol 27:895–905. 10.1016/j.tcb.2017.08.002 28890254 10.1016/j.tcb.2017.08.002 PMC5696061

[CR37] Li R, Wang B, Cao X et al (2022) Sevoflurane exposure in the developing brain induces hyperactivity, anxiety-free, and enhancement of memory consolidation in mice. Front Aging Neurosci 14:934230. 10.3389/fnagi.2022.934230 35847668 10.3389/fnagi.2022.934230 PMC9278137

[CR36] Li L, Meng F, Li D (2023) Downregulation of Nrf2 in the hippocampus contributes to postoperative cognitive dysfunction in aged rats by sensitizing oxidative stress and neuroinflammation. Oxid Med Cell Longev 2023:7272456. 10.1155/2023/7272456 36819786 10.1155/2023/7272456 PMC9935806

[CR35] Li J, Wu Y, Wang Y et al (2025) Activation of glutamatergic neurons in the supramammillary nucleus promotes the recovery of consciousness under Sevoflurane anesthesia. Adv Sci (Weinh Baden-Wurtt Ger) 12:e2406959. 10.1002/advs.202406959 10.1002/advs.202406959 PMC12140388 40167172

[CR39] Liu L, Liu C, Fang L (2021) AMPK–SIRT1 pathway dysfunction contributes to neuron apoptosis and cognitive impairment induced by Sevoflurane. Mol Med Rep 23:1–1. 10.3892/mmr.2020.11694 10.3892/mmr.2020.11694 PMC7706003 33200801

[CR38] Liu A, Zhu X-J, Sun W-D et al (2025) Nicotinamide N-methyltransferase as a potential therapeutic target for neurodegenerative disorders: mechanisms, challenges, and future directions. Exp Neurol 389:115253. 10.1016/j.expneurol.2025.115253 40221009 10.1016/j.expneurol.2025.115253

[CR40] Maejima Y, Yokota S, Yamachi M et al (2025) Oxytocin enhances demethylation through TET enzyme expression in neurons of aged mice: oxytocin as a potential antiaging peptide. Aging Cell E. 10.1111/acel.70198 10.1111/acel.70198 PMC12507420 40788779

[CR41] Mahapatra G, Gao Z, Bateman JR et al (2024) Peripheral blood cells from older adults exhibit sex-associated differences in mitochondrial function. J Gerontol Biol Sci Med Sci 79:glae098. 10.1093/gerona/glae098 10.1093/gerona/glae098 PMC11059251 38602189

[CR42] Marcozzi S, Bigossi G, Giuliani ME et al (2025) A novel cognitive frailty index for geriatric mice. Aging Cell 24:e70056. 10.1111/acel.70056 40395103 10.1111/acel.70056 PMC12266745

[CR43] Meara JG, Leather AJM, Hagander L et al (2015) Global surgery 2030: evidence and solutions for achieving health, welfare, and economic development. Lancet 386:569–624. 10.1016/S0140-6736(15)60160-X 25924834 10.1016/S0140-6736(15)60160-X

[CR44] Müller KAL, Chatterjee M, Rath D, Geisler T (2015) Platelets, inflammation and anti-inflammatory effects of antiplatelet drugs in ACS and CAD. Thromb Haemost 114:498–518. 10.1160/TH14-11-0947 26224127 10.1160/TH14-11-0947

[CR45] Musillo C, Samà M, Creutzberg KC et al (2025) Sex-dependent preventive effects of prenatal N-acetyl-cysteine on neuronal, emotional and metabolic dysfunctions following exposure to maternal high-fat diet in mice. Transl Psychiatry 15:306. 10.1038/s41398-025-03530-0 40846701 10.1038/s41398-025-03530-0 PMC12373772

[CR46] Olejnik P, Golenia A, Maciejewska O et al (2025) Sex-related disparities in cognitive impairment in kidney transplant patients with kidney failure. J Nephrol. 10.1007/s40620-025-02436-w 41139161 10.1007/s40620-025-02436-w PMC12712118

[CR47] Olesen MA, Pradenas E, Villavicencio-Tejo F et al (2024) Mitochondria-tau association promotes cognitive decline and hippocampal bioenergetic deficits during the aging. Free Radical Biol Med 217:141–156. 10.1016/j.freeradbiomed.2024.03.017 38552927 10.1016/j.freeradbiomed.2024.03.017

[CR48] Parrini M, Caroni P, Spolidoro M (2024) Protocol to investigate the gradual selection and deployment of goal-oriented search strategies during unsupervised navigation in mice. STAR Protoc 5:103290. 10.1016/j.xpro.2024.103290 39226172 10.1016/j.xpro.2024.103290 PMC11419921

[CR49] Peña CJ (2025) Epigenetic regulation of brain development, plasticity, and response to early-life stress. Neuropsychopharmacol: Off Publ Am Coll Neuropsychopharmacol. 10.1038/s41386-025-02179-z 10.1038/s41386-025-02179-z PMC12618498 40770493

[CR50] Peng S, Zhao S, Yan F et al (2015) HDAC2 selectively regulates FOXO3a-mediated gene transcription during oxidative stress-induced neuronal cell death. J Neurosci: Off J Soc Neurosci 35:1250–1259. 10.1523/JNEUROSCI.2444-14.2015 10.1523/JNEUROSCI.2444-14.2015 PMC6605534 25609639

[CR51] Petrič B, Kramberger MG, Dolžan V et al (2025) The role of age and sex in paraoxonase 1 activity in patients with alzheimer’s dementia. Chem-Biol Interact. 10.1016/j.cbi.2025.111787 10.1016/j.cbi.2025.111787 41130349

[CR52] Pfeiffer BE (2022) Spatial learning drives rapid goal representation in hippocampal ripples without place field accumulation or goal-oriented theta sequences. J Neurosci: Off J Soc Neurosci 42:3975–3988. 10.1523/JNEUROSCI.2479-21.2022 10.1523/JNEUROSCI.2479-21.2022 PMC9097771 35396328

[CR53] Picard K, Bisht K, Poggini S et al (2021) Microglial-glucocorticoid receptor depletion alters the response of hippocampal microglia and neurons in a chronic unpredictable mild stress paradigm in female mice. Brain Behav Immun 97:423–439. 10.1016/j.bbi.2021.07.022 34343616 10.1016/j.bbi.2021.07.022

[CR54] Qiang Q, Manalo JM, Sun H et al (2021) Erythrocyte adenosine A2B receptor prevents cognitive and auditory dysfunction by promoting hypoxic and metabolic reprogramming. PLOS Biol 19:e3001239. 10.1371/journal.pbio.3001239 34138843 10.1371/journal.pbio.3001239 PMC8211187

[CR55] Reger ML, Hovda DA, Giza CC (2009) Ontogeny of rat recognition memory measured by the novel object recognition task. Dev Psychobiol 51:672–678. 10.1002/dev.20402 19739136 10.1002/dev.20402 PMC2956740

[CR56] Roychowdhury S, Chinnaiyan AM (2016) Translating cancer genomes and transcriptomes for precision oncology. CA Cancer J Clin 66:75–88. 10.3322/caac.21329 26528881 10.3322/caac.21329 PMC4713245

[CR57] Sanacora G, Yan Z, Popoli M (2022) The stressed synapse 2.0: pathophysiological mechanisms in stress-related neuropsychiatric disorders. Nat Rev Neurosci 23:86–103. 10.1038/s41583-021-00540-x 34893785 10.1038/s41583-021-00540-x

[CR58] Santana RA, McWhirt JM, Brewer GJ (2025) Treatment of age-related decreases in GTP levels restores endocytosis and autophagy. GeroScience. 10.1007/s11357-025-01786-4 10.1007/s11357-025-01786-4 PMC12972173 40751793

[CR59] Shen R, Ding Y, Dong Q et al (2024) IL-4-induced gene 1: a potential player in myocardial infarction. Rev Cardiovasc Med 25:337. 10.31083/j.rcm2509337 39355609 10.31083/j.rcm2509337 PMC11440439

[CR60] Shye S, Yanai J, Pick CG (1994) Directional consistency: determinant of learned maze performance of five mice strains. Behav Processes 32:117–131. 10.1016/0376-6357(94)90070-1 24895976 10.1016/0376-6357(94)90070-1

[CR61] Souza IN, de O, Roychaudhuri R, de Belleroche J, Mothet J-P (2023) d-amino acids: new clinical pathways for brain diseases. Trends Mol Med 29:1014–1028. 10.1016/j.molmed.2023.09.001 37770379 10.1016/j.molmed.2023.09.001

[CR62] Srs AB, K M, T P, et al (2025) Sex differences and postoperative cognitive dysfunction: unveiling biological mechanisms. Psychoneuroendocrinology. 10.1016/j.psyneuen.2025.107524 10.1016/j.psyneuen.2025.107524 40561807

[CR63] Sun Z, Shi J, Liu C et al (2024) The effect of low-dose dexmedetomidine on perioperative neurocognitive dysfunction in elderly patients undergoing endoscopic retrograde cholangiopancreatography (ERCP): a randomized, controlled, double-blind trial. Drug Des Devel Ther 18:3715–3725. 10.2147/DDDT.S470514 39193191 10.2147/DDDT.S470514 PMC11348930

[CR64] Tanaka KZ, He H, Tomar A et al (2018) The hippocampal engram maps experience but not place. Sci (n Y NY) 361:392–397. 10.1126/science.aat5397 10.1126/science.aat5397 30049878

[CR65] Tang A, Xu M, Chen X et al (2025) Somatostatin-expressing neurons in the medial prefrontal cortex promote Sevoflurane anesthesia in mice. Anesthesiology 142:844–862. 10.1097/ALN.0000000000005394 39869666 10.1097/ALN.0000000000005394

[CR66] Tao G, Zhang J, Zhang L et al (2014) Sevoflurane induces Tau phosphorylation and glycogen synthase kinase 3β activation in young mice. Anesthesiology 121:510–527. 10.1097/ALN.0000000000000278 24787352 10.1097/ALN.0000000000000278 PMC4165789

[CR67] Vacas S, Kurien P, Maze M (2013) Sleep and anesthesia - common mechanisms of action. Sleep Med Clin 8:1–9. 10.1016/j.jsmc.2012.11.009 28747855 10.1016/j.jsmc.2012.11.009 PMC5524381

[CR68] Varga DK, Raykov PP, Jefferies E et al (2025) Hippocampal mismatch signals are based on episodic memories and not schematic knowledge. Proc Natl Acad Sci U S A 122:e2503535122. 10.1073/pnas.2503535122 40844765 10.1073/pnas.2503535122 PMC12403140

[CR69] Venkateshappa C, Harish G, Mythri RB et al (2012) Increased oxidative damage and decreased antioxidant function in aging human substantia Nigra compared to striatum: implications for parkinson’s disease. Neurochem Res 37:358–369. 10.1007/s11064-011-0619-7 21971758 10.1007/s11064-011-0619-7

[CR70] Wagenhäuser MU, Mulorz J, Krott KJ et al (2024) Crosstalk of platelets with macrophages and fibroblasts aggravates inflammation, aortic wall stiffening, and osteopontin release in abdominal aortic aneurysm. Cardiovasc Res 120:417–432. 10.1093/cvr/cvad168 37976180 10.1093/cvr/cvad168

[CR72] Wang P, Cao J, Liu N et al (2016) Protective effects of Edaravone in adult rats with surgery and lipopolysaccharide administration-induced cognitive function impairment. PLoS ONE 11:e0153708. 10.1371/journal.pone.0153708 27116382 10.1371/journal.pone.0153708 PMC4846078

[CR71] Wang D-S, Ju L, Pinguelo AG et al (2024a) Crosstalk between GABAA receptors in astrocytes and neurons triggered by general anesthetic drugs. Transl Res: J Lab Clin Med 267:39–53. 10.1016/j.trsl.2023.11.007 10.1016/j.trsl.2023.11.007 38042478

[CR73] Wang Z, Dong J, Zhang M et al (2024b) Sevoflurane-induced overexpression of extrasynaptic α5-GABAAR via the RhoA/ROCK2 pathway impairs cognitive function in aged mice. Aging Cell 23:e14209. 10.1111/acel.14209 38825816 10.1111/acel.14209 PMC11488297

[CR74] Woll KA, Zhou X, Bhanu NV et al (2018) Identification of binding sites contributing to volatile anesthetic effects on GABA type A receptors. FASEB J 32:4172–4189. 10.1096/fj.201701347R 29505303 10.1096/fj.201701347R PMC6044061

[CR76] Wu Y, Zhang D, Liu J et al (2024) Activity of the sodium leak channel maintains the excitability of paraventricular thalamus glutamatergic neurons to resist anesthetic effects of Sevoflurane in mice. Anesthesiology 141:56–74. 10.1097/ALN.0000000000005015 38625708 10.1097/ALN.0000000000005015

[CR75] Wu X, He T, He F, Liu L (2025) Is postoperative cognitive dysfunction a disease of microglial inflammatory memory? A state-transition model from metabolic stress to epigenetic lock-in. Front Mol Neurosci 18:1648161. 10.3389/fnmol.2025.1648161 40843245 10.3389/fnmol.2025.1648161 PMC12364888

[CR78] Xia S-F, Xie Z-X, Qiao Y et al (2015) Differential effects of Quercetin on hippocampus-dependent learning and memory in mice fed with different diets related with oxidative stress. Physiol Behav 138:325–331. 10.1016/j.physbeh.2014.09.008 25447470 10.1016/j.physbeh.2014.09.008

[CR77] Xia J-M, Fan B-Q, Yi X-W et al (2024) Medial septal glutamatergic neurons modulate States of consciousness during Sevoflurane anesthesia in mice. Anesthesiology 140:102–115. 10.1097/ALN.0000000000004798 37812765 10.1097/ALN.0000000000004798

[CR79] Xie X, Zhang X, Li S, Du W (2024) Involvement of Fgf2-mediated Tau protein phosphorylation in cognitive deficits induced by Sevoflurane in aged rats. Mol Med 30:39. 10.1186/s10020-024-00784-0 38493090 10.1186/s10020-024-00784-0 PMC10943822

[CR80] Xu Y, Wang X, Xu Z et al (2023) Tbx2 knockdown alleviated sevoflurane-induced cognitive disorder and neuron damages in aged rats via suppressing oxidative stress and ferroptosis. Toxicol Sci 195:257–269. 10.1093/toxsci/kfad071 37494465 10.1093/toxsci/kfad071

[CR83] Yang N-S-Y, Zhong W-J, Sha H-X et al (2024a) mtDNA-cGAS-STING axis-dependent NLRP3 inflammasome activation contributes to postoperative cognitive dysfunction induced by Sevoflurane in mice. Int J Biol Sci 20:1927–1946. 10.7150/ijbs.91543 38481801 10.7150/ijbs.91543 PMC10929193

[CR84] Yang T-T, Wei R, Jin F-F et al (2024b) Dexmedetomidine alleviates the Long-Term neurodevelopmental toxicity induced by Sevoflurane in the developing brain. Dev Neurosci. 10.1159/000542114 10.1159/000542114 PMC12140586 39433029

[CR85] Yang Z-J, Huang S-Y, Zhong K-Y et al (2024c) Betaine alleviates cognitive impairment induced by homocysteine through attenuating NLRP3-mediated microglial pyroptosis in an m6A-YTHDF2-dependent manner. Redox Biol 69:103026. 10.1016/j.redox.2024.103026 38184996 10.1016/j.redox.2024.103026 PMC10808937

[CR82] Yang G, Zhao Y, Zhao C et al (2025) Kv1.1 channel dysfunction in parvalbumin-positive interneurons contributes to anxiety-like behaviors in young adult presenilin 1/2 conditional double knockout mice. Cell Biosci 15:89. 10.1186/s13578-025-01422-w 40563122 10.1186/s13578-025-01422-w PMC12199491

[CR86] Yeung J, Li W, Holinstat M (2018) Platelet signaling and disease: targeted therapy for thrombosis and other related diseases. Pharmacol Rev 70:526–548. 10.1124/pr.117.014530 29925522 10.1124/pr.117.014530 PMC6013590

[CR87] Yin C, Zhang M, Cheng L et al (2024) Melatonin modulates TLR4/MyD88/NF-κB signaling pathway to ameliorate cognitive impairment in sleep-deprived rats. Front Pharmacol 15:1430599. 10.3389/fphar.2024.1430599 39101143 10.3389/fphar.2024.1430599 PMC11294086

[CR88] Zhang L, Cheng Y, Xue Z et al (2022) Sevoflurane impairs m6A-mediated mRNA translation and leads to fine motor and cognitive deficits. Cell Biol Toxicol 38:347–369. 10.1007/s10565-021-09601-4 33928466 10.1007/s10565-021-09601-4

[CR89] Zhang X, Wang L, Zou X et al (2025) Neonatal Sevoflurane anesthesia induces persistent cognitive deficits in mice through CypD-dependent mitochondrial impairment in parvalbumin interneurons. Chem-Biol Interact 420:111700. 10.1016/j.cbi.2025.111700 40784489 10.1016/j.cbi.2025.111700

[CR90] Zhao B, Zhou S, Wei T et al (2025a) Lost in space and thought: navigating the cognitive map in alzheimer’s disease. Neurosci Bull. 10.1007/s12264-025-01541-x 41201576 10.1007/s12264-025-01541-x PMC12950124

[CR91] Zhao X, Chen J, Shan M et al (2025b) Identification of potential IL4I1 inhibitors through structure-based virtual screening and molecular dynamics simulations. J Biomol Struct Dyn. 10.1080/07391102.2025.2501666 10.1080/07391102.2025.2501666 40411435

[CR92] Zheng S, Teng Y, Liu H et al (2024) Syringaresinol attenuates Tau phosphorylation and ameliorates cognitive dysfunction induced by Sevoflurane in aged rats. J Neuropathol Exp Neurol 83:596–605. 10.1093/jnen/nlae026 38622895 10.1093/jnen/nlae026 PMC11187417

[CR93] Zhou J, Zhang C, Fang X et al (2023a) Activation of autophagy inhibits the activation of NLRP3 inflammasome and alleviates sevoflurane-induced cognitive dysfunction in elderly rats. BMC Neurosci 24:9. 10.1186/s12868-023-00777-5 36709248 10.1186/s12868-023-00777-5 PMC9883890

[CR95] Zhou Y, Zhang Y, Wang H et al (2023b) Microglial pyroptosis in hippocampus mediates sevolfurane-induced cognitive impairment in aged mice via ROS-NLRP3 inflammasome pathway. Int Immunopharmacol 116:109725. 10.1016/j.intimp.2023.109725 36764275 10.1016/j.intimp.2023.109725

[CR94] Zhou S, Cui X, Chen J et al (2024) Single exposure to anesthesia/surgery in neonatal mice induces cognitive impairment in young adult mice. Free Radic Biol Med 214:184–192. 10.1016/j.freeradbiomed.2024.02.017 38369077 10.1016/j.freeradbiomed.2024.02.017

[CR96] Zhu Y, Zhang M, Wang J, Wang Q (2022) Lin28A reduced Sevoflurane-Induced nerve injury and cognitive dysfunction by inhibiting Tau acetylation and phosphorylation via activating SIRT1 in elderly rats. Neurotox Res 40:1913–1923. 10.1007/s12640-022-00594-4 36322362 10.1007/s12640-022-00594-4

