# Supplementary material for: Transcriptomic Profiling in Aged Mice Reveals an Association Between Sevoflurane Anesthesia and Neurocognitive Dysfunction
Source: Cell Mol Neurobiol. 2026 Jan 30;46:39. doi: 10.1007/s10571-026-01677-y (PMC12906444; doi:10.1007/s10571-026-01677-y)
Supplement: Supplementary file 4 — Supplementary Material 4 [file 10571_2026_1677_MOESM4_ESM.pdf]

RNA-seq Methods

1. Flow chart of Experiments

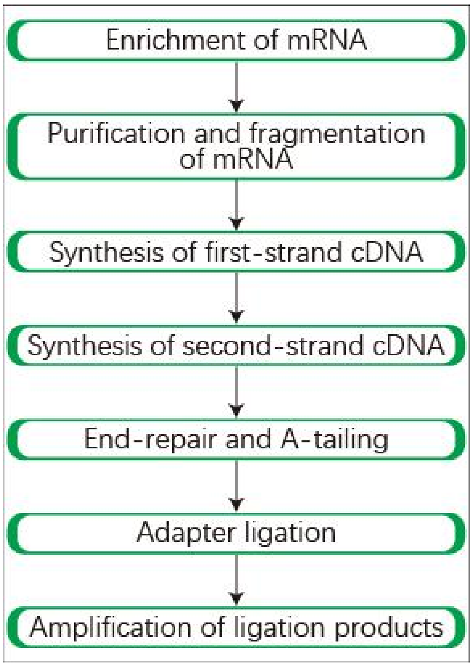

1.1 Library construction and sequencing

The extracted mRNA is enriched using mRNA Capture Beads. After purification with beads, the mRNA is fragmented using high temperatures. The fragmented mRNA is then used as a template to synthesize the first strand of cDNA in a reverse transcription enzyme mixture system. While synthesizing the second strand of cDNA, end repair and A-tailing are completed. Next, adapters are ligated, and Hieff NGS® DNA Selection Beads are used for purification to select target fragments. PCR library amplification is then performed, and finally, detection is carried out using the Illumina Novaseq X Plus.

2. Bioinformatics analysis

2.1 Filtering of Clean Reads

Reads obtained from the sequencing machines includes raw reads containing adapters or low quality bases which will affect the following assembly and analysis. Thus, to get high quality clean reads, reads were further filtered by fastp (version 0.18.0).

The parameters were as follows:

1) removing reads containing adapters;

2) removing reads containing more than 10% of unknown nucleotides(N);

3) removing low quality reads containing more than 50% of low quality (Q-value≤20) bases.

2.2 Alignment with Ribosome RNA (rRNA)

Short reads alignment tool Bowtie2 (version 2.2.8) was used for mapping reads to ribosome RNA (rRNA) database. The rRNA mapped reads then will be removed. The remaining clean reads were further used in assembly and gene abundance calculation.

2.3 Alignment with Reference Genome

An index of the reference genome was built, and paired-end clean reads were mapped to the reference genome using HISAT2 2.1.0 and other parameters set as a default.

2.4 Quantification of Gene Abundance

The mapped reads of each sample were assembled by using StringTie v1.3.1 in a reference-based approach. For each transcription region, a FPKM (fragment per kilobase of transcript per million mapped reads) value was calculated to quantify its expression abundance and variations, using RSEM software.

The FPKM formula is shown as follows:

$$\mathrm{FPKM} = \frac{10^6 \mathrm{C}}{\mathrm{NL}/10^3}$$

Given FPKM(i) to be the expression of gene i, C to be number of fragments mapped to gene i, N to be total number of fragments that mapped to reference genes, and L to be number of bases on gene i. The FPKM method is able to eliminate the influence of different gene lengths and sequencing data amount on the calculation of gene expression. Therefore, the calculated gene expression can be directly used for comparing the difference of gene expression among samples. The mapped reads of each sample were assembled by using StringTie v1.3.1 in a reference-based approach. For each transcription region, a TPM (Transcripts Per Kilobase of exon model per Million mapped reads) value was calculated to quantify its expression abundance and variations, using RSEM software. The TPM formula is shown as follows:

$$TPM = \frac{N_i/L_i \times 10^6}{sum\ (N_1/L_1 + N_2/L_2 + \ldots + N_n/L_n)}$$

Given TPM(i) to be the expression of gene i, N to be reads count of gene i, L to be number of bases on gene i, and sum() to be total number of reads count of all genes after normalization. The TPM method is able to eliminate the influence of different gene lengths and sequencing data amount on the calculation of gene expression. Therefore, the calculated gene expression can be directly used for comparing the difference of gene expression among samples.

2.5 Relationship analysis of samples

2.5.1 Correlation Analysis of Replicas

Correlation analysis was performed by R. Correlation of two parallel experiments provides the evaluation of the reliability of experimental results as well as operational stability. The correlation coefficient between two replicas was calculated to evaluate repeatability between samples. The closer the correlation coefficient gets to 1, the better the repeatability between two parallel experiments.

2.5.2 Principal Component Analysis

Principal component analysis (PCA) was performed with R package gmodels in this experience. PCA is a statistical procedure that converts hundreds of thousands of correlated variables (gene expression) into a set of values of linearly uncorrelated variables called principal components. PCA is largely used to reveal the structure/relationship of the samples/datas.

2.6 Differentially expressed genes (DEGs)

RNAs differential expression analysis was performed by DESeq2 software between two different groups. The genes/transcripts with the parameter of false discovery rate (FDR) below 0.05 (FDR<0.05) and |log2FC|>1were considered differentially expressed genes/transcripts.

2.6.1 GO Enrichment Analysis

Gene Ontology (GO) is an international standardized gene functional classification system which offers a dynamic-updated controlled vocabulary and a strictly defined concept to comprehensively describe properties of genes and their products in any organism. GO has three ontologies: molecular function, cellular component and biological process. The basic unit of GO is GO-term. Each GO-term belongs to a type of ontology.

GO enrichment analysis provides all GO terms that significantly enriched in DEGs comparing to the genome background, and filter the DEGs that correspond to biological functions. Firstly all DEGs were mapped to GO terms in the Gene Ontology database, gene numbers were calculated for every term, significantly enriched GO terms in DEGs comparing to the genome background were defined by hypergeometric test. The calculating formula of P-value is:

$$P = 1 - \sum_{i=0}^{m-1} \frac{\binom{M}{i}\binom{N-M}{n-i}}{\binom{N}{n}}$$

Here N is the number of all genes with GO annotation; n is the number of DEGs in N; M is the number of all genes that are annotated to the certain GO terms; m is the number of DEGs in M. The calculated p-value were gone through FDR Correction, taking FDR ≤ 0.05 as a threshold. GO terms meeting this condition were defined as significantly enriched GO terms in DEGs. This analysis was able to recognize the main biological functions that DEGs exercise.

2.6.2 Pathway Enrichment Analysis

Genes usually interact with each other to play roles in certain biological functions. Pathway-based analysis helps to further understand genes biological functions. KEGG is the major public pathway-related database. Pathway enrichment analysis identified significantly enriched metabolic pathways or signal transduction pathways in DEGs comparing with the whole genome background. The calculating formula is the same as that in GO analysis.

$$P = 1 - \sum_{i=0}^{m-1} \frac{\binom{M}{i}\binom{N-M}{n-i}}{\binom{N}{n}}$$

Here N is the number of all genes that with KEGG annotation, n is the number of DEGs in N, M is the number of all genes annotated to specific pathways, and m is number of DEGs in M. The calculated p-value was gone through FDR Correction, taking FDR ≤ 0.05 as a threshold. Pathways meeting this condition were defined as

significantly enriched pathways in DEGs.
